# Cancer-selective cytotoxic Ca^2+^ overload in acute myeloid leukemia cells and attenuation of disease progression in mice by synergistically acting polyphenols curcumin and carnosic acid

**DOI:** 10.18632/oncotarget.7240

**Published:** 2016-02-12

**Authors:** Stella Pesakhov, Matan Nachliely, Zeev Barvish, Nasma Aqaqe, Bar Schwartzman, Elena Voronov, Yoav Sharoni, George P. Studzinski, Daniel Fishman, Michael Danilenko

**Affiliations:** ^1^ Department of Clinical Biochemistry and Pharmacology, Ben-Gurion University of the Negev, Beer Sheva 84105, Israel; ^2^ The Shraga Segal Department of Microbiology and Immunology, Ben-Gurion University of the Negev, Beer Sheva 84105, Israel; ^3^ Department of Pathology and Laboratory Medicine, Rutgers-New Jersey Medical School, Newark, NJ 07103, USA; ^4^ Department of Physiology and Cell Biology, Ben-Gurion University of the Negev, Beer Sheva 84105, Israel; ^5^ Permanent address: Blood Bank Institute, Soroka University Medical Center, Beer Sheva 85025, Israel; ^6^ Permanent address: Department of Pathology, Sackler Faculty of Medicine Tel-Aviv University, Tel-Aviv 69978, Israel

**Keywords:** acute myeloid leukemia, intracellular calcium, curcumin, carnosic acid, apoptosis

## Abstract

Acute myeloid leukemia (AML) is an aggressive hematologic malignancy characterized by extremely heterogeneous molecular and biologic abnormalities that hamper the development of effective targeted treatment modalities. While AML cells are highly sensitive to cytotoxic Ca^2+^ overload, the feasibility of Ca^2+^- targeted therapy of this disease remains unclear. Here, we show that apoptotic response of AML cells to the synergistically acting polyphenols curcumin (CUR) and carnosic acid (CA), combined at low, non-cytotoxic doses of each compound was mediated solely by disruption of cellular Ca^2+^ homeostasis. Specifically, activation of caspase cascade in CUR+CA-treated AML cells resulted from sustained elevation of cytosolic Ca^2+^ (Ca^2+^_cyt_) and was not preceded by endoplasmic reticulum stress or mitochondrial damage. The CUR+CA-induced Ca^2+^_cyt_ rise did not involve excessive influx of extracellular Ca^2+^ but, rather, occurred due to massive Ca^2+^ release from intracellular stores concomitant with inhibition of Ca^2+^_cyt_ extrusion through the plasma membrane. Notably, the CUR+CA combination did not alter Ca^2+^ homeostasis and viability in non-neoplastic hematopoietic cells, suggesting its cancer-selective action. Most importantly, co-administration of CUR and CA to AML-bearing mice markedly attenuated disease progression in two animal models. Collectively, our results provide the mechanistic and translational basis for further characterization of this combination as a prototype of novel Ca^2+^-targeted pharmacological tools for the treatment of AML.

## INTRODUCTION

Acute myeloid leukemia (AML) is one of the most aggressive hematologic malignancies in adults, with 5-year relative survival rates of 17–19% [1]. The standard approach to treat AML has changed little in the last four decades and employs the cytotoxic drugs cytarabine and anthracyclines. However, while as many as 80% of AML patients may achieve a complete remission after standard chemotherapy (e.g., [2]), relapse is inevitable for the majority of these patients who will ultimately die of the disease. Targeted treatment modalities, such as the use of FLT3 and Polo-like kinase inhibitors, which can be suitable for the elderly, are currently explored for AML therapy [3]. However, tremendous heterogeneity and multitude of molecular aberrations that underlie the hard-to-treat nature of AML [4, 5] hamper the development of such modalities, indicating an urgent need for implementation of alternative strategies for the therapy of this disease.

Cellular Ca^2+^ tightly controls diverse processes, ranging from enhanced proliferation and survival in response to transient elevation of cytosolic Ca^2+^ ([Ca^2+^] _cyt_) to apoptosis triggered by long-term [Ca^2+^] _cyt_ rise [6]. Consequently, interference with cellular Ca^2+^ transport by small molecule inhibitors or silencing of particular Ca^2+^ channels and pumps has been reported to attenuate progression of many types of experimental malignancies [7–10]. Feasibility of Ca^2+^-targeted cancer therapies is illustrated by successful coupling of the plant sesquiterpene lactone thapsigargin, a non-selective Ca^2+^-mobilizing inducer of apoptosis, to masking peptides which undergo specific proteolysis in prostate carcinoma cells and neovasculature of other solid tumors. A resulting prodrug (G202) is currently in clinical trials [7]. AML cells are highly vulnerable to elevation of [Ca^2+^]_cyt_ [11–15], but no therapeutic agents for selective disruption of Ca^2+^ homeostasis in this cancer cell type are currently available.

In addition to thapsigargin, other phytochemicals, e.g., plant polyphenols [16–20], were found to induce apoptosis associated with alteration of Ca^2+^ homeostasis in neoplastic cells, though the exact mechanisms underlying polyphenol-mediated Ca^2+^ changes remain poorly understood. Different polyphenols have been reported to suppress AML in cell-based and murine models [21–25], and some of these compounds, such as curcumin (CUR) and resveratrol, have been in cancer clinical trials [26, 27]. However, low bioavailability of these agents limits their therapeutic application, and the concentrations needed for retardation of tumor growth in rodents greatly exceed doses compatible with human use [27–29]. The utilization of the ability of polyphenols to synergistically enhance anticancer effects of one another in different models [30–33] has been suggested, among other approaches [28, 34], to improve their bioactivity *in vivo*. Using this combination strategy, we have previously shown that CUR and another polyphenol, carnosic acid (CA), combined at potentially bioavailable non-cytotoxic concentrations of each agent robustly triggered apoptosis in KG-1a and HL60 AML cells [35]. Here, we demonstrate a cancer-selective cytotoxicity of the CUR+CA combination in a broad panel of AML cell lines and, importantly, the attenuation of AML progression in CUR+CA-treated mice. Furthermore, we show that a rapid caspase-dependent apoptosis induced by CUR+CA in AML cells is mediated solely by Ca^2+^_cyt_ overload due to massive release of Ca^2+^ from intracellular stores and inhibition of Ca^2+^_cyt_ extrusion through the plasma membrane.

## RESULTS

### The CUR+CA combination produces AML- suppressive effects *in vitro* and *in vivo*

**Figure 1:**
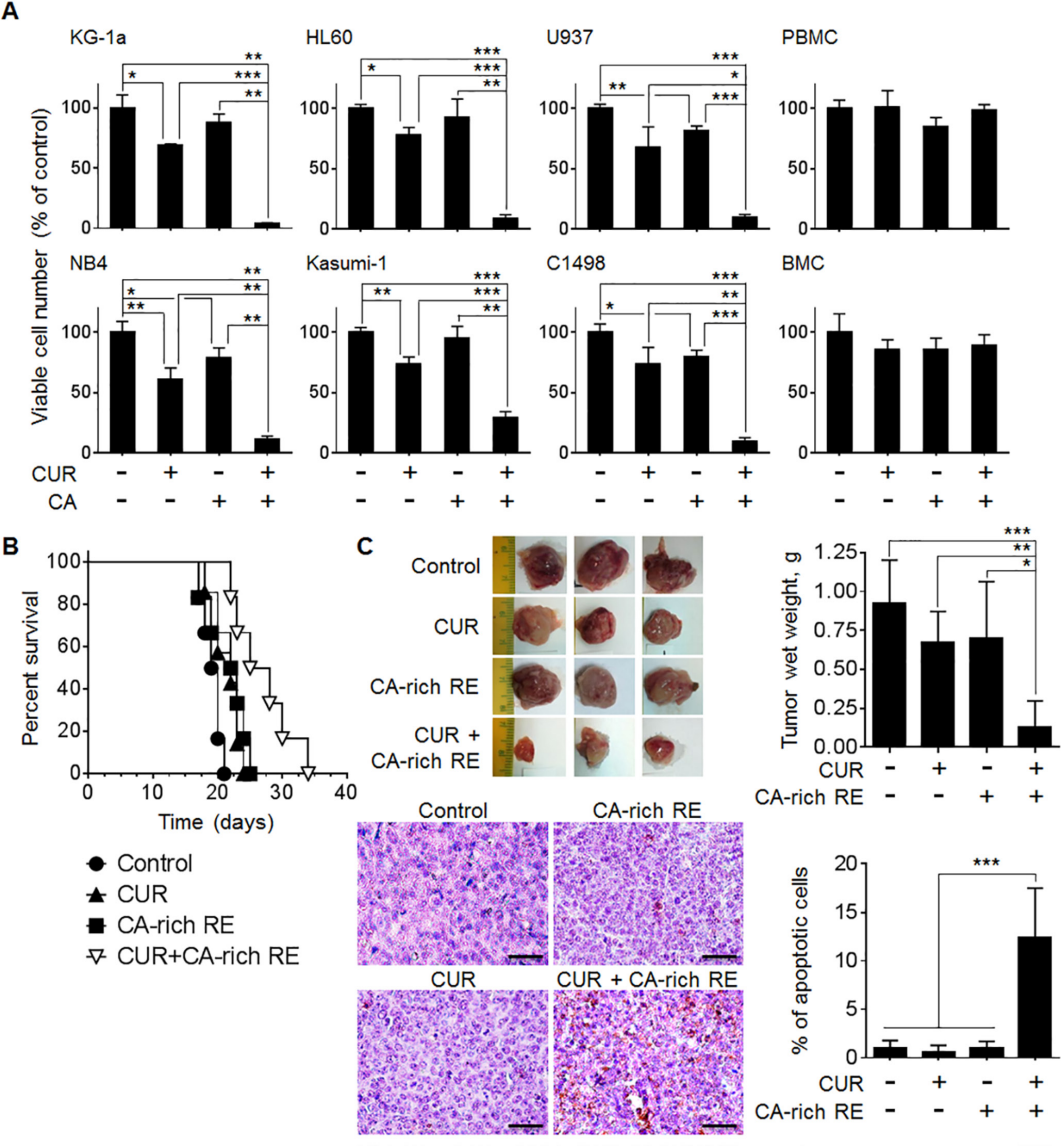
CUR and CA synergistically reduce the numbers of viable AML cells in culture and retard AML development in murine models (**A**) Indicated AML and non-neoplastic hematopoietic cell types were treated with 5 μM CUR and/or 10 μM CA for 72 h. Viable cell numbers were determined by the trypan blue exclusion assay. The data are the means ± SD from 3–6 independent experiments performed in triplicate. (**B**) C57BL/6 mice were inoculated i.v. with 5 × 10^4^ C1498 cells. Seven days thereafter, mice were divided into 4 groups of 6 animals and fed with diets containing vehicle (2.05% methylcellulose, w/w), CUR (0.05%, w/w), CA-rich RE (2%, w/w) or CUR+CA-rich RE. Animal survival was estimated by the Kaplan-Meier analysis. (**C**) SCID/Beige mice were inoculated i.p. with 5 × 10^6^ HL60 cells. Seven days thereafter, mice were divided into 4 groups of 6 animals and treated i.p. with vehicle (0.1% DMSO/PBS), CUR (25 mg/kg), CA-rich RE (25 mg/kg) or CUR+CA-rich RE, 3 times a week for 12 days. *Upper left panel*: typical tumors excised from control and treated animals. *Upper right panel*: Averaged tumor weights (means ± SD) in the indicated groups of mice. *(*p* < 0.05), **(*p* < 0.01), ***(*p* < 0.001); Student's *t* test. *Lower left panel*: TUNEL staining of apoptosis (400×). Scale bars indicate 50 μm. *Lower right panel*: Average percentages of apoptotic cells calculated as described in Materials and Methods. ***(*p* < 0.001); Student's *t* test.

We have previously demonstrated a dose-dependent synergistic cytotoxicity of the CUR+CA combination in HL60 and KG-1a AML cells [35]. To assess translational implications of these findings, we first tested if CUR+CA selectively elicit cytotoxicity in additional AML cell types vs. non-neoplastic hematopoietic cells. CUR and CA were combined at the doses of 5 μM and 10 μM, respectively, at which each agent alone produced modest cytotoxicity in our previous studies [35, 36]. Indeed, singly applied CUR and CA only moderately reduced viable cell counts in cultures of human (U937, NB4 and Kasumi-1) and murine (C1498) AML cell lines, whereas co-application of both agents resulted in a dramatic loss of cell viability (Figure 1A). Notably, the CUR+CA combination did not alter the viability of normal human peripheral blood mononuclear cells (PBMC) and murine bone marrow cells (BMC), supporting specific cytotoxicity of this combination towards AML cells.

The anti-leukemic effects of the CUR+CA combination were then assessed in systemic [37] and the peritoneal leukemic tumor [38] models of AML. To observe a possible cooperation between these agents *in vivo*, we decided to apply relatively lower dosages of CUR and CA-rich rosemary extract (RE) within the dose ranges used in similar animal studies (Supplementary Table 1). Thus, in the systemic model, dietary co-administration of purified CUR (0.05%, w/w) and CA-rich rosemary extract (2%, w/w) to syngeneic C57BL/6 mice intravenously inoculated with C1498 AML cells significantly (*p* = 0.006) prolonged median survival by ~35% compared to untreated control animals which developed fulminant disease about 2 weeks after cell inoculation (Figure 1B). In the local leukemic tumor model generated by i.p. inoculation of HL60 cells into SCID/Beige mice, two out of six animals injected i.p. with the combination of CUR (25 mg/kg) and CA-rich rosemary extract (25 mg/kg) did not develop visible tumors. The rest of the combination-treated mice developed smaller tumors with significantly reduced weights compared to untreated control animals (Figure 1C). Inhibition of tumor progression in these mice was accompanied by a markedly increased extent of apoptosis in malignant tissue, as determined by the TUNEL assay (Figure 1C). In both *in vivo* models, treatment with single agents had only a slight antileukemic effect (Figure 1B, 1C). All treatments were well tolerated since we did not observe significant changes in general habitus, behavior and animal body weight gain in C57BL/6 mice prior to the appearance of leukemia symptoms (Supplementary Figure S1A) or in SCID/Beige mice throughout the experiment (Supplementary Figure S1B). Altogether, these results underscore the prominent capability of CUR and CA to cooperate in producing enhanced antileukemic effects both in cellular and animal models of AML.

### The CUR+CA combination selectively induces apoptosis in AML cells through Ca^2+^-dependent activation of caspases −8 and −9

**Figure 2:**
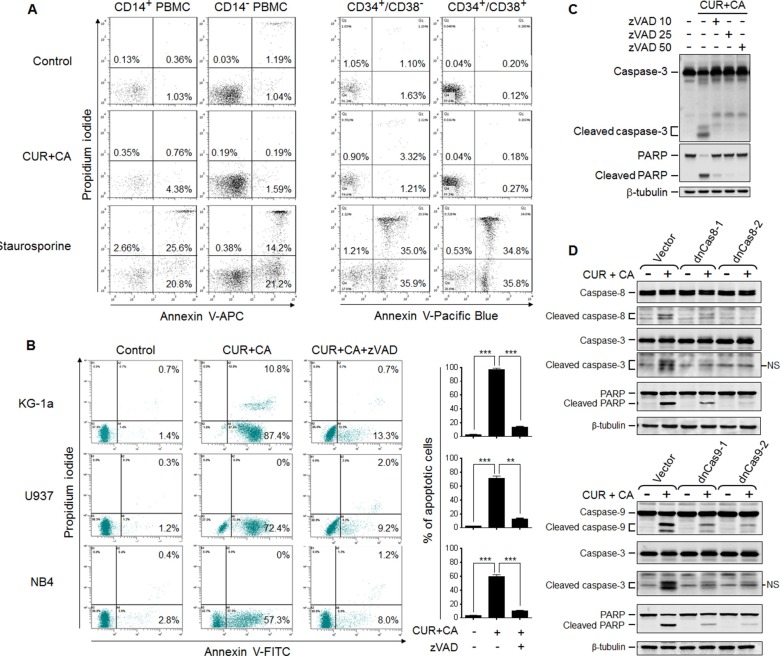
CUR+CA combination induces caspase-dependent apoptosis in AML cells but not in non-neoplastic hematopoietic cells (**A**) PBMC (*left panel*) and umbilical cord blood mononuclear cells (*right panel*) were treated with vehicle (control) or the CUR+CA combination for 18 h, or with 1.25 μM staurosporine (positive control) for 6 h. The extent of apoptosis was determined in the indicated cell populations (Supplementary Figure S2) by flow cytometry using the annexin V/propidium iodide binding assay. Representative panels from at least 3 similar experiments performed in each cell type are shown. (**B**) Indicated AML cell types were treated with the CUR+CA combination and analyzed for apoptotic responses, as above. Note that apoptosis was nearly abolished by the pan-caspase inhibitor zVAD (50 μM). *Left panels*: typical dot plots obtained in a representative experiment. Percentages of early (annexin V-positive, propidium iodide-negative) and late (annexin V- and propidium iodide-positive) cells are shown. *Right panels*: percentages of apoptotic (early+late) cells measured in 3 independent experiments (means ± SD). (**C**) Western blot analysis of caspase-3 and PARP cleavage in KG-1a cells treated with CUR+CA for 7 h in the presence or absence of zVAD. (**D**) Caspase (8, 9 and 3) and PARP cleavage in KG-1a cells stably transfected with the empty vector, the dominant-negative caspase-8- (dnCas8; *upper panel*) or caspase-9- (dnCas9; *lower panel*) encoding plasmids. Cells were treated with or without CUR+CA for 7 h. Two clones of each dnCas8 and dnCas9 transfectant were used. Data from a representative experiment of 3 performed are shown. **(*p* < 0.01), ***(*p* < 0.001); Student's *t* test.

**Figure 3:**
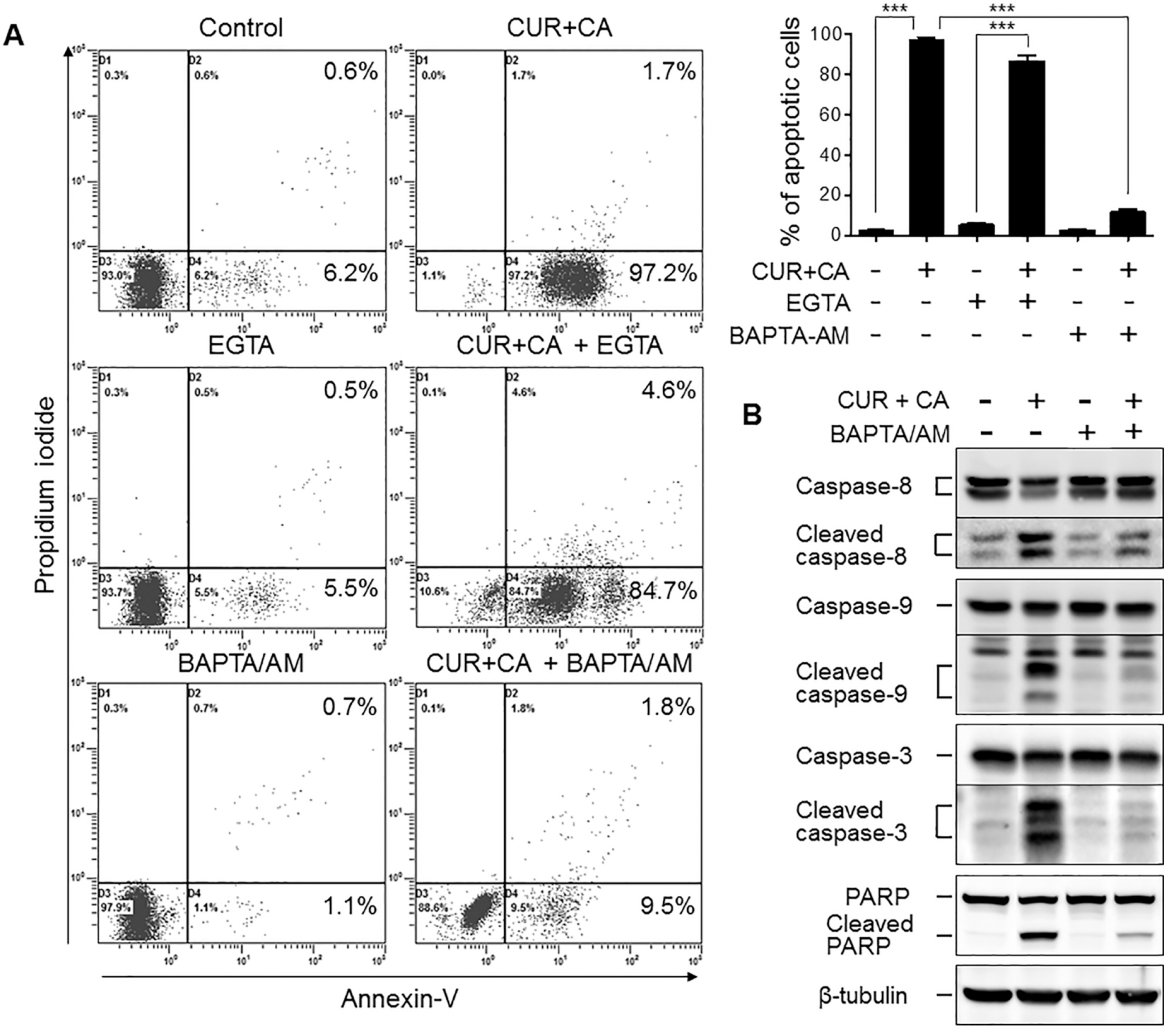
Intracellular but not extracellular Ca^2+^ mediates the CUR+CA-induced apoptosis in AML cells (**A**) Annexin V/propidium iodide assay was used to assess the extent of apoptosis in control and CUR+CA-treated (7 h) KG-1a cells, in the presence or absence of EGTA (1 mM) or BAPTA/AM (2 μM). *Left panel*: typical dot plots obtained in a representative experiment. *Right panel*: averaged percentages of apoptotic (early+late) cells measured in 3 independent experiments (means ± SD). (**B**) Western blot analysis of caspase (8, 9 and 3) and PARP cleavage in control and CUR+CA-treated KG-1a cells (7 h), in the presence or absence of BAPTA/AM (2 μM). ***(*p* < 0.001); Student's *t* test.

To further characterize the cancer-selective cytotoxicity of CUR+CA, we first compared the extent of apoptosis in different types of AML and non-neoplastic hematopoietic cells treated by this combination. Remarkably, no induction of apoptosis was detected in monocytic (larger, CD14-positive cells) and lymphocytic (smaller, CD14-negative cells) populations of CUR+CA-treated non-cycling PBMC (Figure 2A and Supplementary Figure S2A), as well as in BMC (Supplementary Figure S3). A similar lack or apoptotic response was also observed in umbilical cord blood stem/progenitor cell populations (Figure 2A), particularly, the primitive, quiescent CD34^+^/CD38^−^ cells and the more mature, cycling CD34^+^/CD38^+^ progenitor cells (Supplementary Figure S2B). In contrast, CUR+CA treatment led to robust apoptosis in AML cells (Figures 2B, 3A, and Supplementary Figure S4), without induction of differentiation (Supplementary Figure S5). Along with the previously reported lack of apoptotic response to CUR+CA of phytohemagglutinin-stimulated mature PBMC [35], the above results strongly suggest that unlike AML cells, non-malignant hematopoietic cells are insensitive to this combination irrespective of their maturation status and proliferative capacity.

Detailed analysis of CUR+CA-induced apoptosis in AML cells revealed that the appearance of annexin V-positive cells was accompanied by caspase-3 and PARP cleavage (Figure 2C) and that these effects were nearly abolished by the pan-caspase inhibitor zVAD (Figure 2B, 2C). Notably, activation of executor caspase-3 in CUR+CA-treated KG-1a cells was temporally coincident with the activation of both initiator caspases 8 and 9 (Supplementary Figure S6). Expression of catalytically incompetent (dominant negative) mutants of caspases−8 and −9 inhibited CUR+CA-induced activation of caspase-3 and PARP cleavage (Figure 2D). These findings indicated that the simultaneous activation of two initiator caspases is necessary for CUR+CA-induced apoptosis and suggested that a common mediator is responsible for their activation. Since elevation of cytosolic calcium (Ca^2+^_cyt_) levels has been shown to activate caspase−8 and −9 [39–42], we tested the involvement of extracellular and intracellular Ca^2+^ in CUR+CA-induced apoptosis. As depicted in Figure 3A, incubation of KG-1a cells in Ca^2+^-free medium supplemented with the membrane-impermeable Ca^2+^ chelator EGTA did not significantly affect the extent of CUR-CA-induced cell death compared to the samples cultured in the Ca^2+^-containing medium. On the contrary, preincubation with the membrane-permeable Ca^2+^ chelator BAPTA/AM prevented caspase activation and rescued AML cells from apoptosis (Figure 3A, 3B; Supplementary Figure S4), indicating that the cytotoxic response to CUR+CA is mediated by mobilization of Ca^2+^ from intracellular sources.

### Mobilization of intracellular Ca^2+^ is essential but not sufficient for apoptosis induction in CUR+CA-treated AML cells

**Figure 4:**
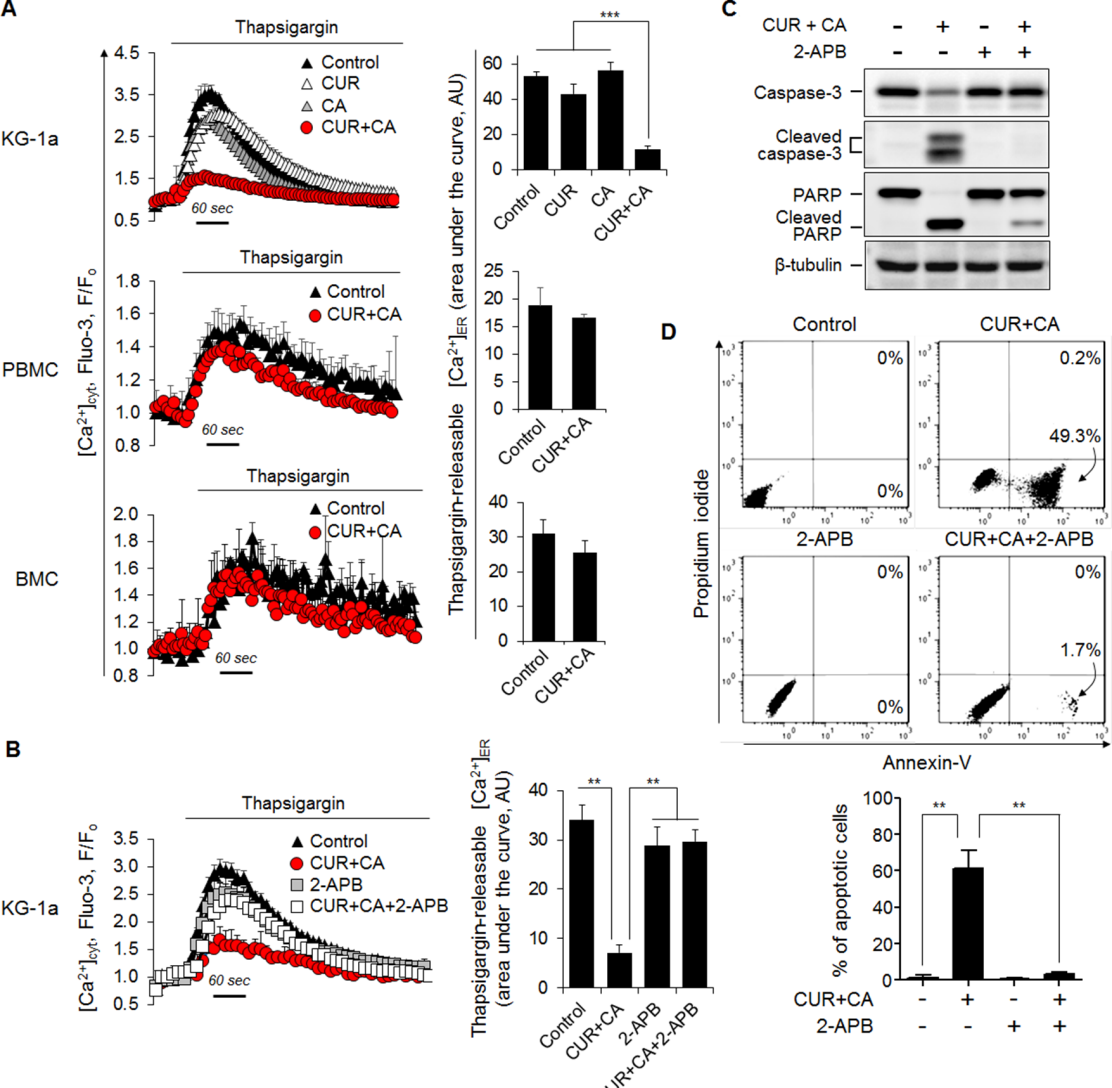
CUR+CA-induced apoptosis is mediated by mobilization of Ca^2+^ from the ER (**A**) CUR+CA treatment depletes thapsigargin-sensitive ER Ca^2+^ stores in AML cells, but not in non-neoplastic hematopoietic cells. *Left panel*: Flow cytometric monitoring of [Ca^2+^]_cyt_ in thapsigargin (1 μM)-stimulated Fluo-3-loaded KG-1a, PBMC and BMC cells following treatment with or without CUR+CA for 2 h. Instantaneous changes in Fluo-3 fluorescence (F) were normalized to the resting values (Fo). Curves show means ± SD of F/Fo ratios measured in a typical experiment. *Right panel*: The extent of thapsigargin-induced Ca^2+^ER release expressed as an area under the curve depicting transient [Ca^2+^]_cyt_ rise followed by [Ca^2+^]_cyt_ decline toward the baseline. The data are the means ± SD from a representative of 3 experiments performed in triplicate. (**B**) Depletion of ER Ca^2+^ stores in CUR+CA-treated KG-1a cells is prevented by the IP3R blocker 2-APB (25 μM). *Left and right* panels show means ± SD of [Ca^2+^]_cyt_ and the extent of thapsigargin-induced Ca^2+^ER release, respectively, measured in a representative of 3 experiments performed in triplicate. (**C**) Western blot analysis of caspase-3 and PARP cleavage in control and CUR+CA-treated KG-1a cells (7 h) in the presence or absence of 2-APB (25 μM). (**D**) Annexin V/propidium iodide assay was used to assess the extent of apoptosis in control and CUR+CA-treated (7 h) KG-1a cells in the presence or absence of 2-APB. *Upper panel*: dot plots from a typical experiment. *Lower panel*: averaged percentages of apoptotic (early+late) cells measured in 3 independent experiments performed in triplicate (means ± SD). **(*p* < 0.01), ***(*p* < 0.001); Student's *t* test.

**Figure 5:**
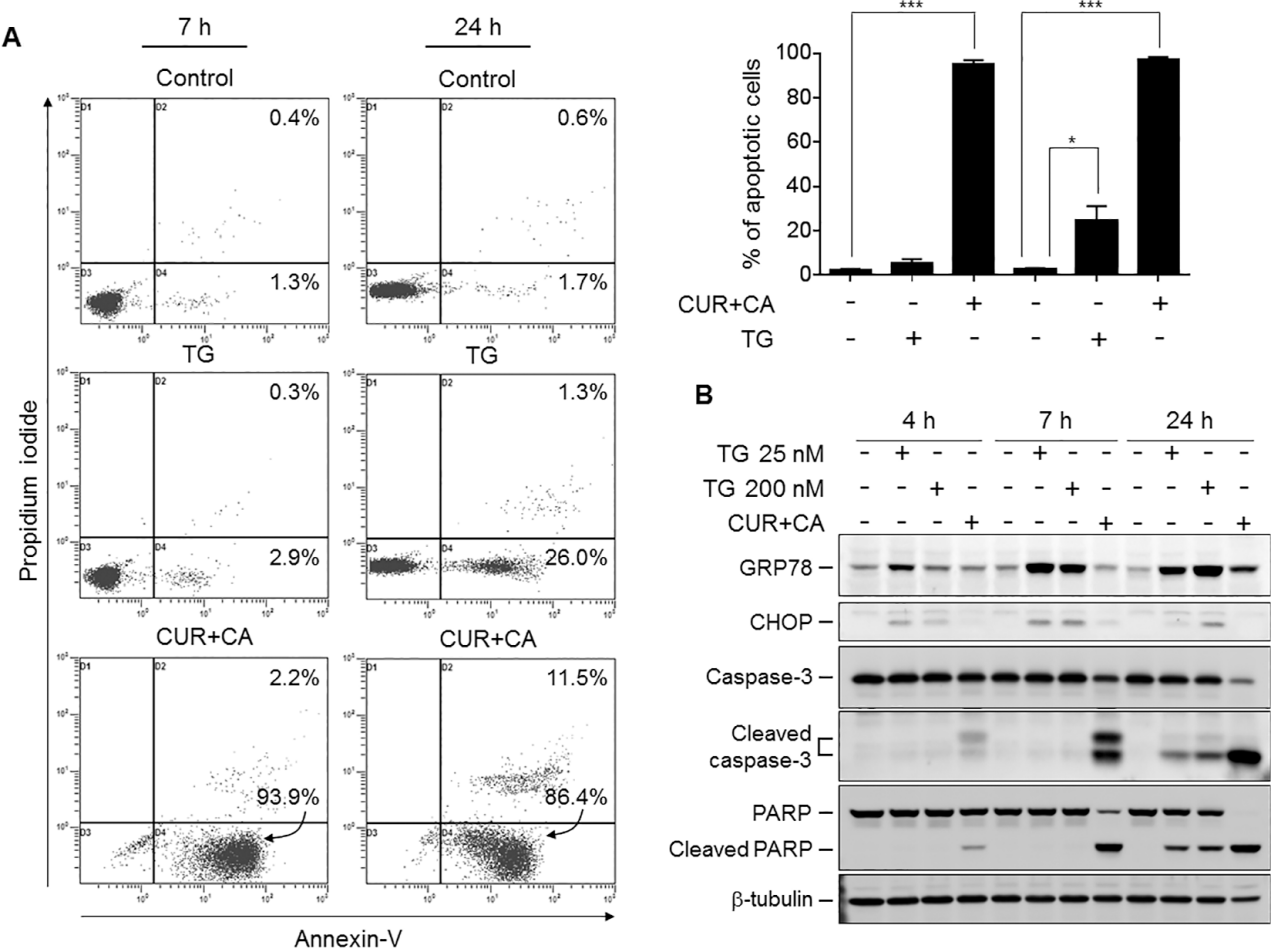
CUR+CA-induced apoptosis is not dependent solely on mobilization of Ca^2+^_ER_ and does not involve ER stress (**A**) Comparison of thapsigargin (200 nM)- and CUR+CA-induced apoptosis in KG-1a cells at the indicated time points using the annexin V/propidium iodide binding assay. *Upper panel*: dot plots from a typical experiment. *Lower panel*: averaged percentages of apoptotic (early+late) cells measured in 3 independent experiments (means ± SD). (**B**) Western blot analysis of the expression of ER stress markers (GRP78; CHOP) and caspase-3 and PARP cleavage in the course of thapsigargin and CUR+CA treatment of KG-1a cells. *(*p* < 0.05), ***(*p* < 0.001); Student's *t* test.

Since ER constitutes the main intracellular Ca^2+^ store, we assessed the amount of ER-releasable Ca^2+^ by the addition of the sarco/endoplasmic reticulum Ca^2+^-ATPase (SERCA) inhibitor thapsigargin (TG) and monitoring the cytosolic Ca^2+^ (Ca^2+^_cyt_) changes. Indeed, CUR+CA treatment for 2 h substantially depleted ER Ca^2+^ stores in these but not in non-neoplastic cells (Figure 4A), as determined by flow cytometry using the fluorescent Ca^2+^_cyt_ indicator Fluo-3-AM [10]. Blockade of ER Ca^2+^ release by the inositol trisphosphate receptor (IP_3_R) Ca^2+^ channel inhibitor 2-aminoethoxydiphenyl borate (2-APB) prevented CUR+CA-induced store depletion (Figure 4B), caspase-3 activation (Figure 4C) and apoptosis (Figure 4D). Notably, depletion of ER Ca^2+^ stores by TG also triggered apoptosis which, however, was evident only after prolonged incubation (24 h) and preceded by up-regulation of the ER stress markers GRP78 and CHOP (Figure 5A, 5B). In contrast, the earlier onset of cell death in CUR+CA-treated cultures was not associated with elevated levels of GRP78 and CHOP, pointing out to different mechanisms of TG- and CUR+CA-induced cell death. These results suggested that mobilization of Ca^2+^ from ER *per se* is important but not sufficient for Ca^2+^-dependent apoptosis in CUR+CA-treated AML cells.

### CUR+CA elicit apoptotic response in AML cells through concerted dysregulation of intracellular Ca^2+^ mobilization and clearance pathways

**Figure 6:**
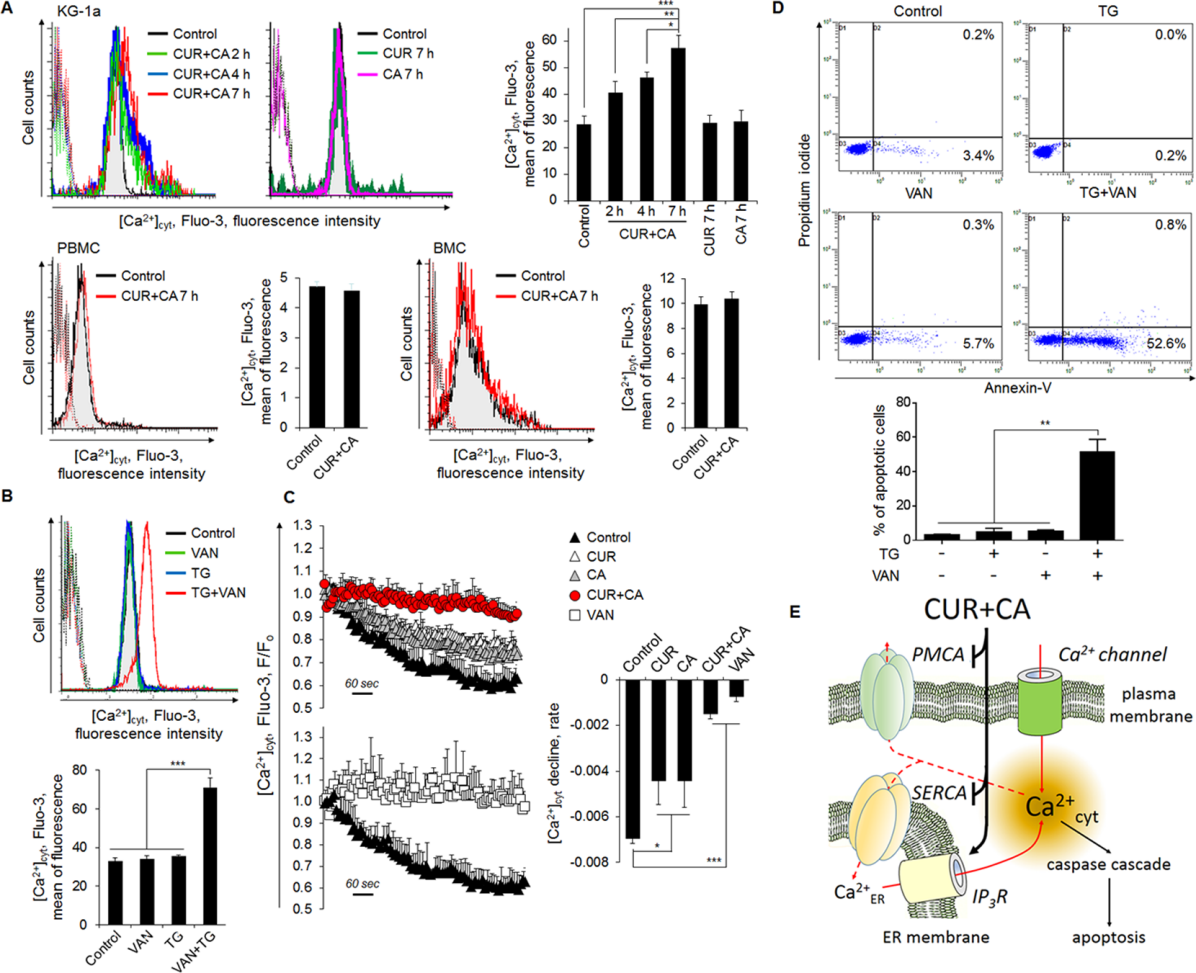
Compromised extrusion of Ca^2+^_cyt_ through the plasma membrane contributes to sustained Ca^2+^_cyt_ rise in CUR+CA-treated AML cells and, thereby, to apoptosis (**A**) CUR+CA treatment elevates steady-state Ca^2+^_cyt_ levels in KG-1a cells but not in PBMC or BMC. [Ca^2+^]_cyt_ is expressed as the mean Fluo-3 fluorescence intensity measured by flow cytometry. Presented are histogram overlays from a typical experiment showing [Ca^2+^]_cyt_ at the indicated time points of CUR+CA treatment. Overlapping dotted curves in the first logarithmic decade depict autofluorescence. Bar graphs show averaged mean Fluo-3 fluorescence intensities ± SD measured in a representative of 3 independent experiments performed in triplicate. (**B**) [Ca^2+^]_cyt_ was assessed in KG-1a cells treated with thapsigargin (TG; 200 nM) and/or the PMCA blocker sodium orthovanadate (VAN; 1 mM) for 7 h, as described in (A). (**C**) CUR+CA treatment blocks Ca^2+^_cyt_ extrusion through the plasma membrane. Fluo-3-labeled KG-1a cells were loaded with Ca^2+^ and extrusion of Ca^2+^_cyt_ was assessed by flow cytometry as the time-dependent decline in Fluo-3 fluorescence, as described in *Materials and Methods*. VAN was used as a positive control for inhibition of plasma membrane Ca^2+^_cyt_ efflux. Curves show means ± SD of F/Fo ratios from a representative of 3 experiments performed in triplicate. Bar graphs show mean F/Fo ratios ± SD. (**D**) Modeling of CUR+CA-induced apoptosis by combined treatment of KG-1a cells with TG (200 nM) and VAN (1 mM) for 7 h. Apoptosis was assessed by the annexin-V/PI binding assay. *Upper panel*: typical dot plots from a representative of 3 independent experiments. Lower panel: averaged percentages of apoptotic (early+late) cells measured in 3 independent experiments (means ± SD). (**E**) A pictorial view for the proposed mechanism of CUR+CA-induced apoptotic Ca^2+^_cyt_ overload in AML cells. Low resting [Ca^2+^]_cyt_ is maintained through the action of SERCA and PMCA. By mobilizing Ca^2+^_ER_ (most probably, via the blockade of SERCA and stimulation of IP_3_R) and inhibiting PMCA, the CUR+CA combination robustly elevates Ca^2+^_cyt_ levels that triggers caspase-dependent apoptosis. *(*p* < 0.05), **(*p* < 0.01), ***(*p* < 0.001); Student's *t* test.

Based on the above results, we proposed that the apoptotic response to CUR+CA results from sustained elevation of Ca^2+^_cyt_ due to compromised clearance of ER-mobilized Ca^2+^. Indeed, a rise in a steady-state Ca^2+^_cyt_ in CUR+CA-treated KG-1a cells, but not in non-neoplastic cells, was detected (Figure 6A). This rise became evident after 2 h, preceding the appearance of apoptotic markers, and increased with time, whereas CUR or CA alone did not change a steady-state Ca^2+^_cyt_ compared to untreated control samples (Figure 6A). CUR has been previously shown to inhibit SERCA and ER Ca^2+^ uptake [43]. However, it seems unlikely that Ca^2+^_cyt_ rise in CUR+CA-treated cells resulted from disruption of the ER Ca^2+^_cyt_ clearance pathway since the SERCA blocker TG was incapable of inducing cytotoxic Ca^2+^_cyt_ overload (Figure 6B). We therefore, tested the possible role of the other major clearance routes, i.e. uptake of Ca^2+^_cyt_ by the mitochondria and its extrusion by the plasma membrane Ca^2+^-ATPase (PMCA). Using the fluorescent mitochondrial Ca^2+^ (Ca^2+^_mit_) indicator Rhod2 and flow cytometric analysis, we did not detect significant differences in the rates of TG-induced mitochondrial Ca^2+^ uptake between CUR+CA-treated and control cells (Supplementary Figure S7). To assess the function of PMCA, Fluo-3-loaded cells were kept on ice and rates of Ca^2+^_cyt_ decline were determined after stimulation of pump activity by heating cells to 37°C [44]. As depicted in Figure 6C, application of either CUR or CA alone markedly reduced the rate of Ca^2+^_cyt_ decline and this inhibitory effect was enhanced by the combined treatment. The results suggested that inhibition of PMCA cooperates with mobilization of ER Ca^2+^ to produce sustained Ca^2+^_cyt_ rise in CUR+CA-treated cells. Indeed, co-application of the potent PMCA inhibitor sodium orthovanadate (VAN, 1 mM) and TG (200 nM) for 7 h mimicked the Ca^2+^_cyt_-elevating (Figure 6B) and apoptotic actions of CUR+CA (Figure 6D). Similar to TG, VAN alone failed to elevate Ca^2+^_cyt_ (Figure 6B) or trigger apoptosis in KG-1a cells (Figure 6D). Collectively, our data underscore a novel mechanism that enables preferential killing of AML cells through concerted dysregulation of ER Ca^2+^ release and Ca^2+^_cyt_ clearance by CUR and CA combined at their low concentrations.

## DISCUSSION

The principal and novel finding of this study is selective targeting of cellular Ca^2+^ homeostasis by the CUR+CA combination in AML cells *vs.* non-neoplastic hematopoietic cells. We have previously reported that when combined at their minimally effective concentrations, these polyphenols synergistically induced a cytotoxic response in KG-1a and HL60 AML cell lines but not PBMC and skin fibroblasts [35]. Our present results obtained in these and additional AML and non-malignant hematopoietic cell types as well as in two murine models suggest the generality of the antileukemic action of the CUR+CA combination in highly heterogeneous AML. Furthermore, we delineate the mechanism underlying the cytotoxic action of this combination in which robust mobilization of Ca^2+^_ER_ cooperates with defective Ca^2+^_cyt_ clearance, to produce Ca^2+^_cyt_ overload and, eventually, apoptotic cell death (Figure 6E).

The involvement of Ca^2+^_cyt_ rise in the apoptotic response to CUR and other polyphenols has previously been reported [16–20]. In contrast to the present research, however, the majority of these studies employed 6-15-fold higher concentrations of CUR [16, 18, 19] and demonstrated an association of Ca^2+^_cyt_ changes with generalized cellular stress events, such as oxidative stress [18, 19], ER stress [18, 20] or mitochondrial damage [16, 19, 20]. We found that none of the above events mediates the cytotoxic response of AML cells to CUR+CA, as supported by our previous and current data. First, no elevation of the intracellular ROS levels or glutathione depletion has been detected following CUR+CA treatment [35]. Second, CUR+CA-induced caspase and PARP cleavage was not preceded by up-regulation of the ER stress markers GRP78 and CHOP (Figure 5B). Third, mitochondrial function was unlikely to be impaired prior to the appearance of apoptotic markers, as reflected by virtually intact mitochondrial Ca^2+^ uptake (Supplementary Figure S7) and mitochondrial membrane potential (Supplementary Figure S8). It is also noteworthy that the mode of sustained Ca^2+^_cyt_ rise in CUR+CA-treated AML cells is distinct from those proposed for polyphenols. Specifically, elevation of Ca^2+^_cyt_ did not involve the previously reported modulation of store-operated [45] or voltage-dependent [46] Ca^2+^ entry because the removal of extracellular Ca^2+^ did not affect the extent of CUR+CA-induced apoptosis (Figure 3A). Likewise, accumulation of Ca^2+^_cyt_ did not result solely from massive Ca^2+^_ER_ release despite the fact that CUR is a known inhibitor of SERCA [43]. The effects of CUR+CA on Ca^2+^ homeostasis and apoptosis induction were reproduced only if the SERCA blocker TG was co-applied with the PMCA inhibitor VAN (Figure 6A, 6B, 6D), indicating that both mobilization of Ca^2+^_ER_ and compromised plasma membrane clearance of Ca^2+^_cyt_ are needed for CUR+CA-induced Ca^2+^_cyt_ rise. It was logical to assume that while CUR inhibits SERCA, CA interferes with PMCA function to elicit this synergistic action. However in our hands, when added separately, each polyphenol produced a similar small reduction in Ca^2+^_ER_ content (Figure 4A) and a moderate inhibition of Ca^2+^_cyt_ extrusion (Figure 6C), suggesting that these compounds act on the same Ca^2+^_ER_-controlling and Ca^2+^_cyt_-extruding cellular systems, and potentiate the activities of each other.

Clearly, a number of questions regarding the mechanism of the synergistic cytotoxic activity of CUR and CA warrant further investigation. For instance, it yet remains to be determined whether this synergy results from chemical stabilization or facilitated intracellular accumulation of one agent by the other due to their antioxidant properties or capabilities to block plasma membrane drug efflux pathways [47, 48]. Another unresolved issue is an apparent selectivity of this combination towards AML cells *vs.* non-malignant hematopoietic cells, which may be related to differential expression of key Ca^2+^ transport proteins in highly malignant cells *vs.* their less malignant or non-neoplastic counterpart cells (See [6] for a recent review). Particularly, a prevalence of SERCA2b over SERCA3 has been reported in non-differentiated *vs.* differentiated AML [49], colon carcinoma [50] and gastric carcinoma [51] cells. Interestingly, quantitative real time PCR analysis conducted in the present study also demonstrated significantly higher SERCA2b/SERCA3 mRNA expression ratios in KG1a and U937 cells compared to PBMC (Supplementary Figure S9), suggesting that SERCA2b may have a role in the selective cytotoxicity of CUR+CA in AML cells. The recently reported involvement of SERCA2b in the apoptotic effects of CUR on human SW872 liposarcoma cells [52] and SKOV3 ovarian cancer [53] cells further supports this suggestion. With regard to other Ca^2+^-controlling proteins, overexpression of Ca^2+^_ER_-mobilizing IP_3_R subtype III but not subtype I has been identified in colon carcinoma [54] and glioblastoma [55] lesions relative to corresponding non-cancerous tissues. Colon and gastric carcinoma cells also showed a higher PMCA1-to-PMCA4 ratio than normal intestinal epithelium [56], while increased levels of PMCA2 have been detected in breast carcinoma specimens and cell lines [57]. Further studies are, therefore, needed to determine which Ca^2+^-regulatory proteins are specifically targeted by the CUR+CA combination in AML cells to elicit the apoptotic response. Irrespective to the above unresolved mechanistic issues, our findings suggest that the CUR+CA combination represents a prototype tool for an alternative mode of Ca^2+^-targeted therapy which triggers cytotoxic Ca^2+^_cyt_ overload in AML but not in non-neoplastic hematopoietic cells.

Indeed, the results of our animal experiments provide a clear perspective regarding the medical relevance of the synergistic AML-suppressive action of the CUR+CA combination. Although the capability of CUR, as a single agent, to retard growth of various experimental malignancies is widely recognized, the clinical use of this polyphenol is limited by its low bioavailability. Consequently, attenuation of tumor development in preclinical studies requires administration of CUR at high doses that cannot be applied to patients [58]. Among these studies, only one has so far demonstrated inhibition of AML cell growth in mice by CUR (100 mg/kg) administered by i.p. injections [21]. However, the i.p. treatment is not a preferable drug delivery route in humans, whereas oral administration of CUR to cancer patients, even at its highest daily safe doses of 8−12 g, has not yet been reported to consistently retard tumor growth [59]. In the present study, not only did we obtain a significant *in-vivo* antileukemic effect by administering i.p. a relatively low dose of CUR (25 mg/kg) together with CA-rich rosemary extract (Figure 1C) but also when combining both agents in the diet (Figure 1B) at concentrations which are in the safe range [37, 38, 59–61].

AML remains a devastating and mostly incurable disease with limited treatment options [1, 62]. Therefore, the observed *in vitro* and *in vivo* synergy between low doses of CUR and CA provides a rationale and justification for further translational and clinical evaluation of this combination for AML therapy and/or prevention. The preventative potential of CUR+CA may be explored in groups at risk for the development of AML which include patients with other malignancies previously treated with cytotoxic and genotoxic agents and individuals with myelodysplastic syndromes [63, 64]. Interestingly, CUR and CA were recently reported to synergistically inhibit the growth of breast cancer cells [65], suggesting that this combination may also have a clinical significance in solid cancers. The fact that plant preparations containing CUR or CA have been widely used as traditional dietary remedies supports the feasibility of oral treatment with their combinations at clinically achievable doses.

## MATERIALS AND METHODS

### Materials

Curcumin was from Cayman Chemicals (Ann Arbor, MI, USA). Carnosic acid was purchased from Enzo Life Sciences (Farmingdale, NY, USA). Standardized carnosic acid-rich (33.9%, w/w) extract of rosemary leaves (lot # LR-06-1-3/A) was kindly provided by LycoRed (Beer Sheva, Israel). MEBCYTO Apoptosis Kit (FITC) and zVAD-fmk were purchased from MBL (Nagoya, Japan). Anti-Annexin V-APC, 1,2-bis(o-aminophenoxy) ethane-N, N, N^#^N^#^-tetraacetic acid tetra-(acetocymethyl)-ester (BAPTA/AM) and 2-aminoethoxydiphenyl borate (2-APB) were purchased from Calbiochem, Merck Biosciences (Schwalbach, Germany). Thapsigargin, sodium orthovanadate, Histopaque-1077, propidium iodide, RNase, and calcium ionophore A23187 were from Sigma (Rehovot, Israel). Fluo-3/AM and Rhod2/AM were purchased from Santa Cruz Biotechnology (Dallas, TX, USA) and TefLabs (Austin, TX, USA), respectively. G418 was from Gibco-Invitrogen (Carlsbad, CA, USA). RPMI 1640 medium, Ca^2+^/Mg^2+^-free phosphate buffered saline (PBS), penicillin, streptomycin, and HEPES were purchased from Biological Industries (Beth Haemek, Israel). Heat-inactivated fetal bovine serum (FBS) was from Gibco-Invitrogen (Carlsbad, CA, USA).

### Plasmids

The pcDNA3.1 construct was purchased from Invitrogen (Carlsbad, CA, USA). Plasmids encoding for catalytically incompetent mutants of caspase 8 (pcDNA3-Casp8 C360A; Addgene plasmid #11818) and caspase-9 (pcDNA3-Casp9 C287A-His; Addgene plasmid #11819) were kindly provided by Dr. G. Salvesen (Sanford-Burnham Medical Research Institute, La Jolla, CA, USA).

### Cell culture

Human KG-1a (CCL-246.1), HL60 (CCL-240), Kasumi-1 (CRL-2724), U937 (CRL-1593.2), and murine C1498 (TIB-49) AML cells were purchased from American Type Culture Collection (Rockville, MD). Human NB-4 (ACC-207) AML cells were purchased from German Collection of Cell Culture (Branschweig, Germany). Samples of peripheral blood and umbilical cord blood were collected with informed consent from healthy adult donors and after full-term deliveries, respectively, upon the approval by the institutional Helsinki committee (Soroka University Medical Center, Beer Sheva, Israel). Mononuclear blood cells were isolated as described previously [35], using Histopaque-1077 gradient centrifugation. To prepare murine bone marrow cells, healthy C57BL/6 mice were humanely sacrificed by the institutionally approved method (CO_2_ suffocation), and bone marrows were removed by repetitive flushing of femurs with ice-cold PBS and re-suspended in a complete growth medium. AML cells were cultured in RPMI 1640 medium supplemented with 10% FBS, penicillin (100 U/ml), streptomycin (0.1 mg/ml), and 10 mM HEPES (pH = 7.4) in a humidified atmosphere of 95% air and 5% CO_2_, at 37°C. Non-neoplastic hematopoietic cells were maintained under the same conditions, except that 20% FBS was used.

### Cell enumeration and viability assay

Cells (4 – 8 × 10^4^/ml) were plated in 24-well plates and incubated with CUR and CA, alone or in combination, for the indicated periods of time. Cell numbers and viability were estimated on the basis of trypan blue exclusion by counting in a Vi-Cell XR cell viability analyzer (Beckman Coulter Inc., Fullerton, CA).

### Assessment of apoptosis by annexin V and propidium iodide staining

Cells were stained using the MEBCYTO^Ò^ Apoptosis Kit (MBL) in accordance with the protocol provided by the manufacturer. Percentages of apoptotic cells were determined by flow cytometry using a Cytomics FC500 instrument (Beckman Coulter, Miami, FL). Ten thousand events were acquired for each sample and the data were analyzed with CXP software (Beckman Coulter).

### Stable transfection

Cell transfections were carried out using the Neon transfection kit (Invitrogen) in accordance with the manufacturer's protocol. Briefly, KG-1a cells (1.5 × 10^5^) were suspended in 10 μl buffer R containing 1 μg of pcDNA3-Cas8 or pcDNA3-Cas9 constructs or pcDNA3.1 empty vector and electroporated (1200 volts, 20 ms, 2 pulses) using a MP-100 microporator (NanoEnTek, Seoul, Korea). Transfected cells were selected by culturing in medium supplemented with 1.8 mg/ml G418. Individual clones were isolated by the limited dilution technique.

### Western blot analysis

Western blotting was performed using whole cell extracts, as described before [35]. Briefly, equal amounts of protein (30 μg) were separated by SDS-PAGE and electroblotted into nitrocellulose membrane (Whatman, Dassel, Germany). The membranes were blocked with 5% milk in Tris-buffered saline containing 0.5% Tween 20 (TBST) for 2 h and incubated with primary antibodies overnight at 4°C. The following primary antibodies were used: caspase-3 (Santa Cruz Biotechnology; cat. #sc-7148, 1:500), caspase-8 (Cell Signaling Technology; cat. #9746; 1:500), caspase-9 (Cell Signaling; cat. #9502; 1:500), poly(ADP-ribose) polymerase (PARP) (Enzo; cat. #BML-SA253, 1:2000), GADD 153 (CHOP) (Santa Cruz; cat. #sc-575, 1:500), GRP78 (Santa Cruz, cat. #sc-13968, 1:500). Blots were washed and incubated with horse-radish peroxidase-conjugated anti-rabbit (Promega, Madison, WI, USA) or anti-mouse (ImmunoResearch Laboratories, West Grove, PA, USA) secondary antibodies. The protein bands were visualized using the Western Lightning™ Chemiluminescence Reagent Plus (PerkinElmer Life Sciences, Inc., Boston, MA). Each membrane was stripped and reprobed for β-tubulin (Santa Cruz, cat. #sc-9104, 1:500), as the internal loading control.

### Measurements of cytosolic Ca^2+^ ([Ca^2+^]_cyt_) by flow cytometry

For evaluation of steady state [Ca^2+^]_cyt_, cells (6 × 10^5^/ml) were suspended in Ca^2+^-supplemented (2 mM) Ringer's solution [10] containing 2.5 μM Fluo-3/AM and 0.1% bovine serum albumin (BSA). Cells were incubated in the dark for 30 min at room temperature, washed by centrifugation, re-suspended in Ca^2+^-supplemented Ringer's solution and analyzed on a FACSCanto instrument (BD Bioscience). Data were processed using FlowJo software, and [Ca^2+^]_cyt_ was expressed as the mean Fluo-3 fluorescence intensity.

For evaluation of endoplasmic reticulum Ca^2+^ (Ca^2+^_ER_) content, cells were loaded with Fluo-3/AM, as described above, and re-suspended in Ca^2+^-free Ringer's solution. Fluo-3 fluorescence was monitored before (resting values) and after the addition of the sarco/endoplasmic reticulum ATPase (SERCA) inhibitor thapsigargin (1 μM). Data were processed with FlowJo software and exported to Microsoft Excel, followed by normalization of the instantaneous changes in Fluo-3 fluorescence to the resting values. The extent of thapsigargin-induced Ca^2+^_ER_ release was determined using Kaleidagraph program (Synergy Software, Reading, PA, USA) and expressed as the area under the curve that depicts a transient [Ca^2+^]_cyt_ rise after the addition of thapsigargin followed by [Ca^2+^]_cyt_ decline toward the baseline.

The Ca^2+^_cyt-_extruding function of the plasma membrane Ca^2+^ ATP-ase (PMCA) was assessed as described [44] with modifications. Namely, all samples were pre-treated with 1 μM thapsigargin for 10 min before the assay to inhibit Ca^2+^_cyt_ uptake by the ER. Fluo-3/AM labeling was performed, as described above. Cells (1.8 × 10^6^) were suspended in 0.3 ml Ringer's solution containing 25 μM CaCl_2_ and incubated at 37°C for 5 min. Ca^2+^ ionophore A23187 was added to a final concentration of 800 nM and suspension was incubated at 37°C for an additional 30 sec to achieve complete Ca^2+^ loading of the cells. A23187 was then removed by transferring cell suspension to 2.7 ml of ice-cold Ringer's solution containing 5 mg/ml BSA, 25 μM CaCl_2_ and, in some samples, 1 mM sodium orthovanadate (PMCA inhibitor) [44]. Samples were maintained on ice before PMCA stimulation by heating to 37°C. Fluo-3 fluorescence was monitored as a function of time, data processed using FlowJo software, exported to Excel and normalized, as described above for Ca^2+^_ER_ measurements. Rates of Ca^2+^_cyt-_extrusion were expressed as slopes of initial 30-sec curve fragment after beginning of [Ca^2+^]_cyt_ decline.

### Mouse models of AML

Experiments were carried out in the Ben-Gurion University SPF animal facility in accordance with the protocol (IL-35-05-2012) approved by the University Committee for the Ethical Care and Use of Animals in Experiments.

For the systemic AML model [37], twenty-four C57BL/6 mice (7 week old; Harlan Laboratories, Ein Kerem, Israel) fed a standard powdered rodent diet (Altromin 1321; Altromin Spezialfutter, Lage, Germany) were inoculated i.v. with 5 × 10^4^ syngeneic C1498 AML cells in 100 μl PBS. Seven days later, animals were randomly assigned to four groups (6 mice/group) and were started on the following supplements mixed with the standard diet (*ad libitum*): Group 1 (vehicle) - 2.05% (w/w) methylcellulose (Sigma); Group 2 – 0.05% (w/w) CUR + 2% (w/w) methylcellulose; Group 3 – 2% (w/w) CA-rich rosemary extract (RE) + 0.05% (w/w) methylcellulose; Group 4 – 0.05% (w/w) CUR + 2% (w/w) CA-rich RE. Animal appearance and behavior were then assessed daily and the body weight was measured 3 days per week. Moribund mice were humanely sacrificed and overall animal survival was estimated by the Kaplan-Meier analysis, as described previously [37].

For the peritoneal AML tumor model [38], twenty-four SCID/Beige mice (6−8 week old; Harlan) fed a pelleted Altromin 1321 diet were inoculated i.p. with 5 × 10^6^ HL60 cells in 200 μl PBS. Seven days later, mice were randomly assigned to four groups (6 mice/group) and were started on the following treatments (200 μl, i.p.): Group 1 (vehicle) – 0.1% DMSO in PBS; Group 2 – 0.5 mg CUR in DMSO/PBS; Group 3 – 0.5 mg CA-rich RE in DMSO/PBS; Group 4 – 0.5 mg CUR + 0.5 mg CA-rich RE in DMSO/PBS. Mice were monitored for another 12 days. Animal appearance and behavior were assessed daily and the body weight was measured every 3 days. At the end of the experiment, all mice were humanely sacrificed and necropsied. Abdominal tumors were excised, weighed and then fixed in 10% neutral buffered formalin and embedded in paraffin.

### TUNEL assay of apoptosis in tumor sections

For the TUNEL assay, tissue sections were deparaffinized and apoptosis was assessed by the terminal deoxynucleotidyl transferase dUTP nick end labeling (TUNEL) assay using ApopTag Peroxidase *In Situ* Apoptosis Detection Kit (Merck Millipore, Billerica, MA) according to the manufacturer's protocol. Slides were counterstained with hematoxylin for visualization of the nuclei. An average percentage of apoptotic cells was calculated after scoring a total of 200 cells in each of 17 random fields at 400× magnification.

### Statistical analysis

The significance of the differences between the means of various subgroups was assessed by unpaired two-tailed Student's *t* test. Animal survival data were statistically evaluated using a log-rank (Mantel-Cox) test. The statistical analysis was performed with GraphPad Prism 6.0 Program (Graph-Pad Software, San Diego, CA). Data are presented as the mean ± SD. *P* < 0.05 was considered statistically significant.

## SUPPLEMENTARY MATERIAL FIGURES AND TABLE


