# Supplementary material for: Cancer-selective cytotoxic Ca2+ overload in acute myeloid leukemia cells and attenuation of disease progression in mice by synergistically acting polyphenols curcumin and carnosic acid
Source: Oncotarget. 2016 Feb 12;7(22):31847–61. doi: 10.18632/oncotarget.7240 (PMC5077981; doi:10.18632/oncotarget.7240)
Supplement: Supplementary file 1 [file oncotarget-07-31847-s001.pdf]

# Cancer-selective cytotoxic $Ca^{2+}$ overload in acute myeloid leukemia cells and attenuation of disease progression in mice by synergistically acting polyphenols curcumin and carnosic acid

## Supplementary Materials

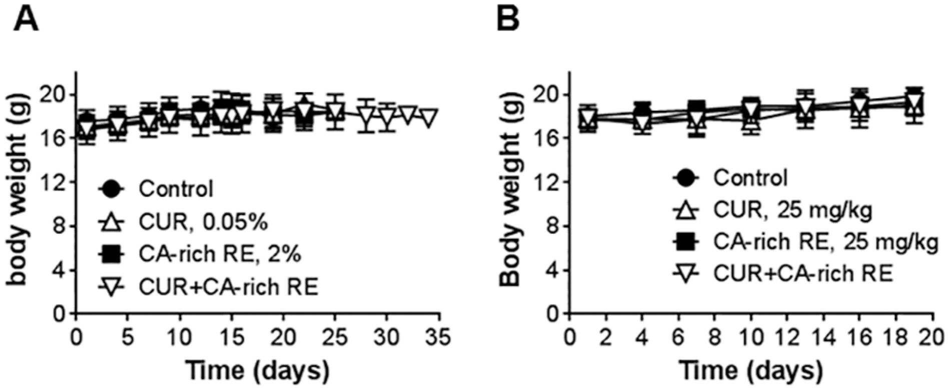


**Supplementary Figure S1: Changes in the animal body weight during treatments with CUR and/or CA-rich rosemary extract.** (**A**) Systemic AML model; (**B**) Peritoneal AML tumor model. The data are the means ± SD ($n$ = 6).

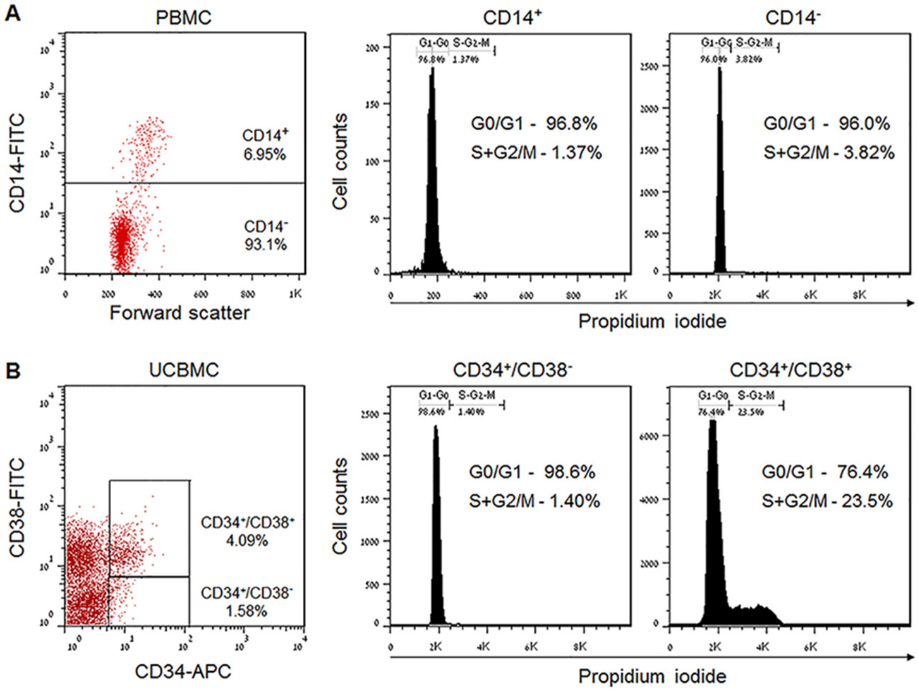


**Supplementary Figure S2: Determination of hematopoietic cell surface markers and cell cycle distribution in PBMC.** (**A**) and umbilical cord blood mononuclear cells (UCBMC) (**B**). (A, B) *Left panels* depict gating strategy for identification of cell populations, in which cell cycle distribution (*right panels*) and the extent of apoptosis (Figure 2A) were analyzed by flow cytometry (Supplementary Materials and Methods). (B, *right panel*) Note that, similar to previously reported data (Supplementary Reference [1]), ~ 25% of $CD34^+$/$CD38^+$ cells are in the proliferation-associated phases (S + G2/M). Representative panels from 3 independent experiments performed in each cell type are shown.

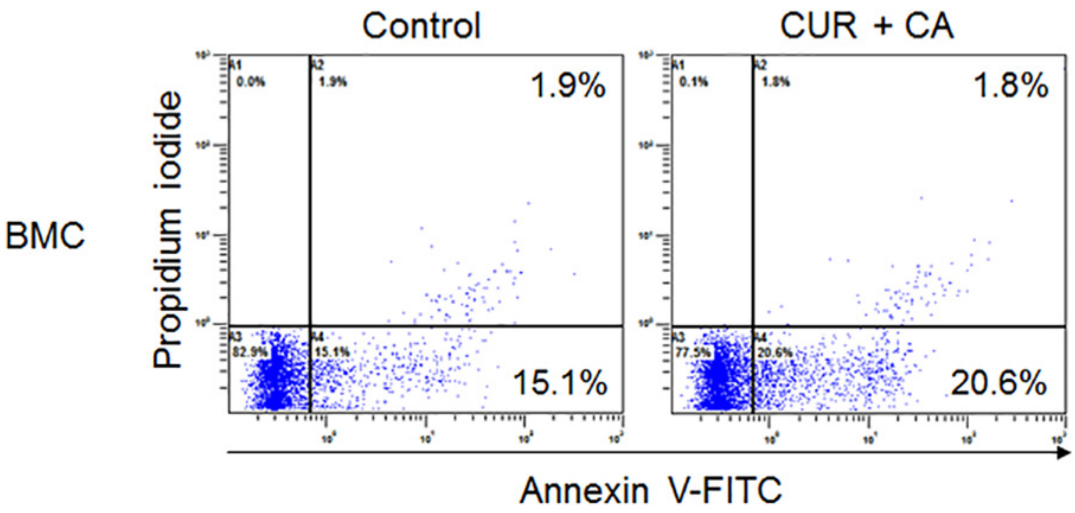


**Supplementary Figure S3: CUR+CA treatment does not induce apoptosis in BMC.** Cells were incubated with vehicle (control) or the CUR+CA combination for 18 h followed by flow cytometric analysis of apoptosis, as described in legend to Figure 2. Representative panels from 3 independent experiments are shown.

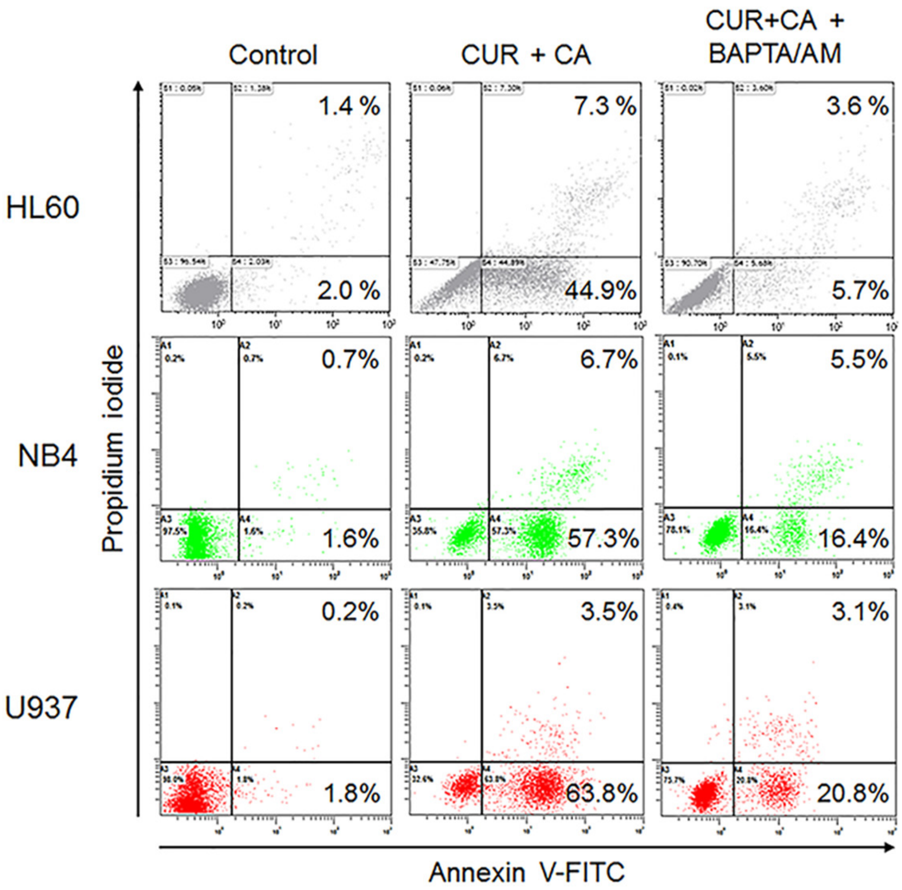


**Supplementary Figure S4: Membrane-permeable $Ca^{2+}$ chelator BAPTA/AM attenuates CUR+CA-induced apoptosis in AML cells.** Cells were treated for 7 h, as described in the legend to Figure 4, and the extent of apoptosis was measured by flow cytometry. Representative dot plots from 1 of 3 experiments in each cell line are shown.

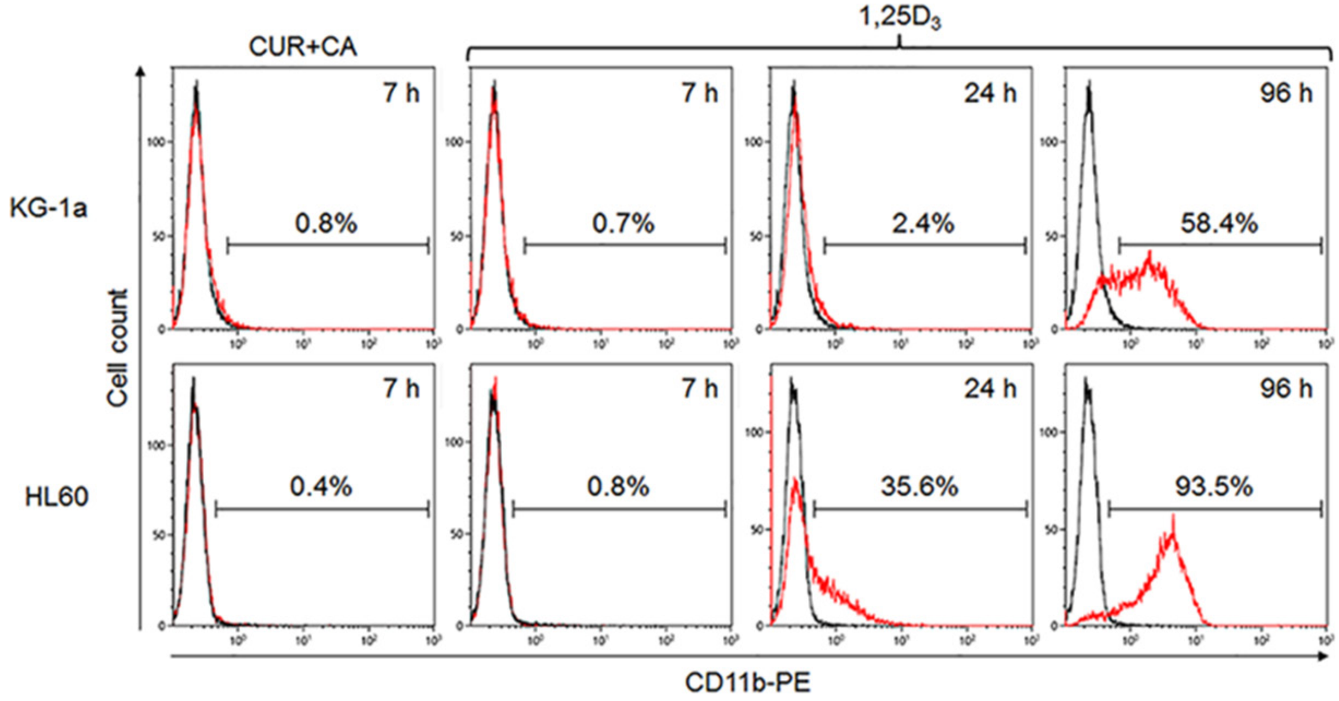


**Supplementary Figure S5: CUR+CA treatment does not induce the CD11b myeloid marker expression in AML cells undergoing apoptosis.** KG-1a and HL60 cells were treated with vehicle or CUR+CA for 7 h following the flow cytometric determination of CD11b expression (Materials and Methods). Note that the potent inducer of myeloid differentiation 1,25-dihydroxyvitamin $D_3$ (the positive control; 100 nM) failed to upregulate CD11b at 7 h, indicating that this time interval is not sufficient for the induction of differentiation in these AML cells.

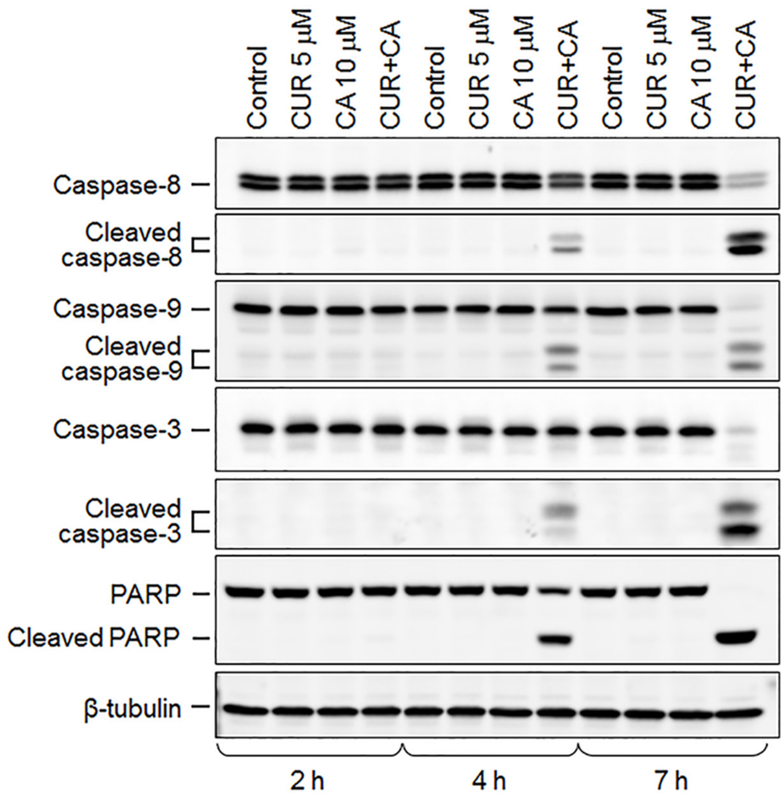


**Supplementary Figure S6: Both initiator caspases (8 and 9) undergo activation in the course of CUR+CA treatment of AML cells.** Western blot analysis of caspase (8, 9 and 3) and PARP cleavage in KG-1a cells at the indicated time points. Representative blots from 3 independent experiments are shown.

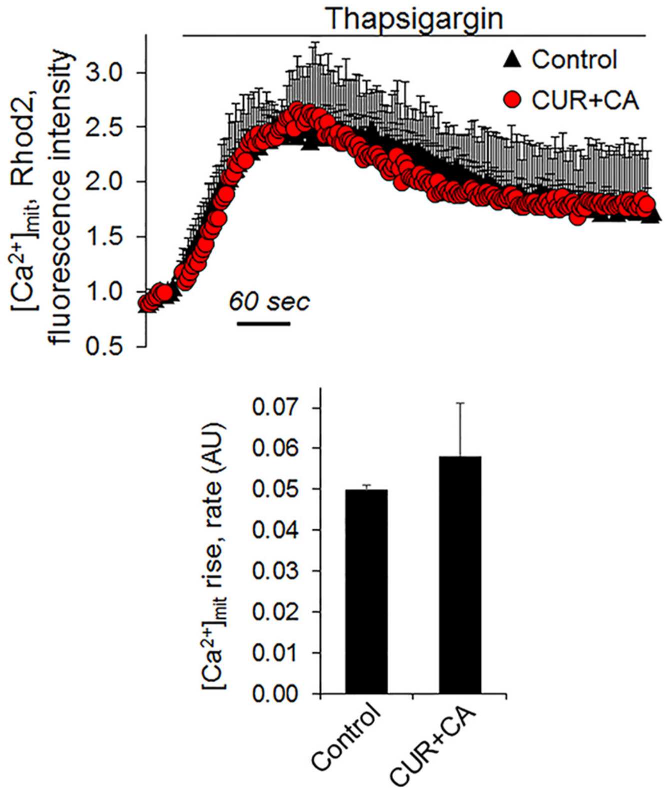


**Supplementary Figure S7: CUR+CA treatment does not alter mitochondrial $Ca^{2+}$ uptake.** Monitoring of $[Ca^{2+}]_{mit}$ in Rhod2-loaded control and CUR+CA-treated KG-1a cells by flow cytometry following stimulation with 10 µM thapsigargin. Instantaneous changes in Rhod2 fluorescence (F) were normalized to the resting values (Fo). *Upper panel*: Curves show means ± SD of F/Fo ratios measured in a representative of 3 experiments performed in triplicate. *Lower panel*: Rates of $Ca^{2+}$ uptake by the mitochondria are expressed as slopes of initial 30-sec curve fragments after beginning of rise in $[Ca^{2+}]$mit. Mean F/Fo ratios ± SD are shown.

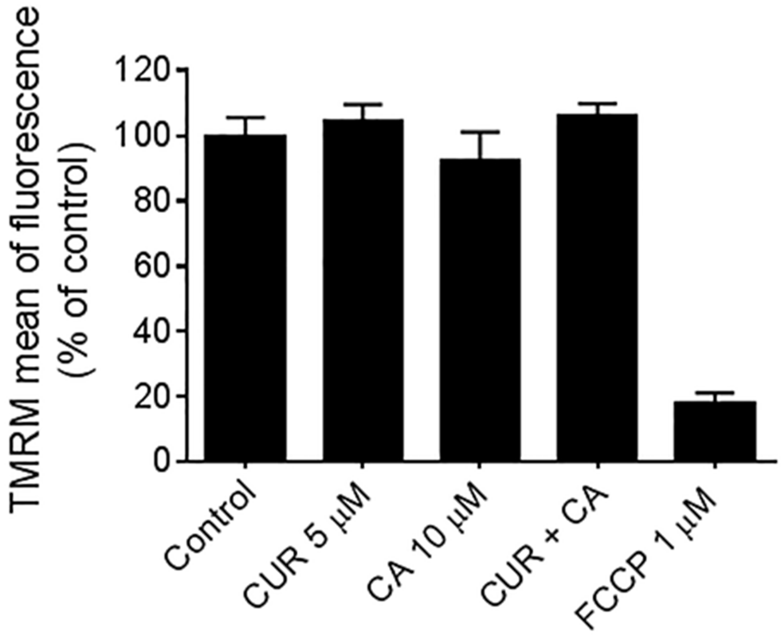


**Supplementary Figure S8: KG-1a cells treated with CUR and/or CA for 2 h retain intact mitochondrial membrane potential (ΔΨm).** Control and treated cells were loaded with tetramethylrhodamine methyl ester (TMRM; 100 nM) for 30 min in the dark, washed and suspended in serum-free medium. Changes in ΔΨm were assesses by flow cytometry. A sample treated with the uncoupler carbonyl cyanide-4-(trifluoromethoxy)phenylhydrazone (FCCP; 1 µM) for 5 min was used as the positive control. The data show averaged means of fluorescence ± SD (as percent of untreated control) from 3 independent experiments performed in triplicate.

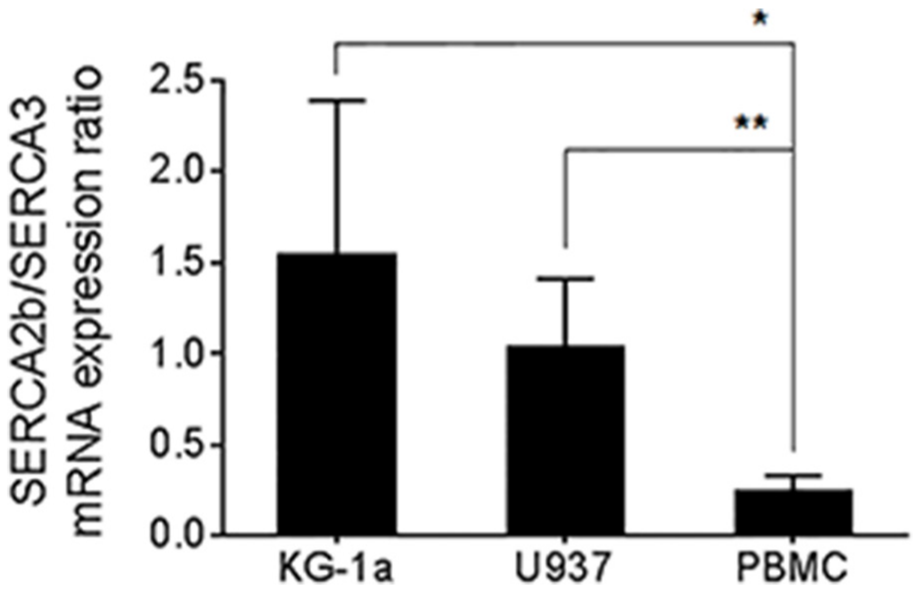


**Supplementary Figure S9: Relative mRNA expression of SERCA2b and SERCA3 isoforms in AML cells and PBMC.** Total RNA was isolated from untreated KG-1a and U937 cells, and PBMC derived from blood samples of two healthy adults (Supplementary Materials and Methods). Bars represent the means ± SD of ΔCt (SERCA2b-GUSB)/ΔCt (SERCA3-GUSB) ratios measured in 4 experiments performed in triplicate. *($p < 0.05$), **($p < 0.01$); Student's *t* test.

**Supplementary Table S1: Dosages of curcumin and rosemary extracts used for dietary and parenteral administration in mouse models of cancer**

| Treatment agent | Administration route | Dose range | References |
|---|---|---|---|
| Curcumin | Dietary | 0.05–1 % (w/w food) | [1–8] |
| | | 2–4 % (w/w food) | [7–12] |
| | Intraperitoneal | 5–25 mg/kg body wt | [13–17] |
| | | 30–60 mg/kg body wt | [9, 18–24] |
| | | 100–120 mg/kg body wt | [19, 22, 25–27] |
| CA-RE | Dietary | 1–4% (w/w food) | [28, 29] |

Abbreviations: CUR, curcumin; CA-RE, carnosic acid-rich rosemary extract.

## REFERENCES


1. Weissenberger J, Priester M, Bernreuther C, Rakel S, Glatzel M, Seifert V, Kogel D. Dietary curcumin attenuates glioma growth in a syngeneic mouse model by inhibition of the JAK1,2/STAT3 signaling pathway. Clin Cancer Res. 2010; 16:5781–5795.
2. Swamy MV, Citineni B, Patlolla JM, Mohammed A, Zhang Y, Rao CV. Prevention and treatment of pancreatic cancer by curcumin in combination with omega-3 fatty acids. Nutr Cancer. 2008; 60 Suppl 1:81–89.
3. Lev-Ari S, Starr A, Katzburg S, Berkovich L, Rimmon A, Ben-Yosef R, Vexler A, Ron I, Earon G. Curcumin induces apoptosis and inhibits growth of orthotopic human non-small cell lung cancer xenografts. J Nutr Biochem. 2014; 25:843–850.
4. Villegas I, Sanchez-Fidalgo S, de la Lastra CA. Chemo-preventive effect of dietary curcumin on inflammation-induced colorectal carcinogenesis in mice. Mol Nutr Food Res. 2011; 55:259–267.
5. Limtrakul P, Lipigorngoson S, Namwong O, Apisariyakul A, Dunn FW. Inhibitory effect of dietary curcumin on skin carcinogenesis in mice. Cancer Lett. 1997; 116:197–203.
6. Bachmeier B, Nerlich AG, Iancu CM, Cilli M, Schleicher E, Vene R, Dell'Eva R, Jochum M, Albini A, Pfeffer U. The chemopreventive polyphenol Curcumin prevents hematogenous breast cancer metastases in immunodeficient mice. Cell Physiol Biochem. 2007; 19:137–152.
7. Barve A, Khor TO, Hao X, Keum YS, Yang CS, Reddy B, Kong AN. Murine prostate cancer inhibition by dietary phytochemicals—curcumin and phenyethylisothiocyanate. Pharm Res. 2008; 25:2181–2189.

8. Huang MT, Lou YR, Ma W, Newmark HL, Reuhl KR, Conney AH. Inhibitory effects of dietary curcumin on forestomach, duodenal, and colon carcinogenesis in mice. Cancer Res. 1994; 54:5841–5847.
9. Tu SP, Jin H, Shi JD, Zhu LM, Suo Y, Lu G, Liu A, Wang TC, Yang CS. Curcumin induces the differentiation of myeloid-derived suppressor cells and inhibits their interaction with cancer cells and related tumor growth. Cancer Prev Res (Phila). 2012; 5:205–215.
10. Aggarwal BB, Shishodia S, Takada Y, Banerjee S, Newman RA, Bueso-Ramos CE, Price JE. Curcumin suppresses the paclitaxel-induced nuclear factor-kappaB pathway in breast cancer cells and inhibits lung metastasis of human breast cancer in nude mice. Clin Cancer Res. 2005; 11:7490–7498.
11. Dorai T, Cao YC, Dorai B, Buttyan R, Katz AE. Therapeutic potential of curcumin in human prostate cancer. III. Curcumin inhibits proliferation, induces apoptosis, and inhibits angiogenesis of LNCaP prostate cancer cells in vivo. Prostate. 2001; 47:293–303.
12. Dahmke IN, Backes C, Rudzitis-Auth J, Laschke MW, Leidinger P, Menger MD, Meese E, Mahlknecht U. Curcumin intake affects miRNA signature in murine melanoma with mmu-miR-205-5p most significantly altered. PLoS One. 2013; 8:e81122.
13. Tung YT, Chen HL, Lai CW, Shen CJ, Lai YW, Chen CM. Curcumin reduces pulmonary tumorigenesis in vascular endothelial growth factor (VEGF)-overexpressing transgenic mice. Mol Nutr Food Res. 2011; 55:1036–1043.
14. Zhou DH, Wang X, Yang M, Shi X, Huang W, Feng Q. Combination of low concentration of (−)-epigallocatechin gallate (EGCG) and curcumin strongly suppresses the growth of non-small cell lung cancer in vitro and in vivo through causing cell cycle arrest. Int J Mol Sci. 2013; 14:12023–12036.
15. Toden S, Okugawa Y, Buhrmann C, Nattamai D, Anguiano E, Baldwin N, Shakibaei M, Boland CR, Goel A. Novel Evidence for Curcumin and Boswellic Acid-Induced Chemoprevention through Regulation of miR-34a and miR-27a in Colorectal Cancer. Cancer Prev Res (Phila). 2015; 8:431–443.
16. Wu SY, Lee YR, Huang CC, Li YZ, Chang YS, Yang CY, Wu JD, Liu YW. Curcumin-induced heme oxygenase-1 expression plays a negative role for its anti-cancer effect in bladder cancers. Food Chem Toxicol. 2012; 50:3530–3536.
17. Fu Z, Chen X, Guan S, Yan Y, Lin H, Hua ZC. Curcumin inhibits angiogenesis and improves defective hematopoiesis induced by tumor-derived VEGF in tumor model through modulating VEGF-VEGFR2 signaling pathway. Oncotarget. 2015; 6:19469–19482.
18. Su CC, Yang JS, Lu CC, Chiang JH, Wu CL, Lin JJ, Lai KC, Hsia TC, Lu HF, Fan MJ, Chung JG. Curcumin inhibits human lung large cell carcinoma cancer tumour growth in a murine xenograft model. Phytother Res. 2010; 24:189–192.
19. Perry MC, Demeule M, Regina A, Moumdjian R, Beliveau R. Curcumin inhibits tumor growth and angiogenesis in glioblastoma xenografts. Mol Nutr Food Res. 2010; 54:1192-1201.
20. Tsai JR, Liu PL, Chen YH, Chou SH, Cheng YJ, Hwang JJ, Chong IW. Curcumin Inhibits Non-Small Cell Lung Cancer Cells Metastasis through the Adiponectin/NF-kappab/MMPs Signaling Pathway. PLoS One. 2015; 10:e0144462.
21. Khor TO, Keum YS, Lin W, Kim JH, Hu R, Shen G, Xu C, Gopalakrishnan A, Reddy B, Zheng X, Conney AH, Kong AN. Combined inhibitory effects of curcumin and phenethyl isothiocyanate on the growth of human PC-3 prostate xenografts in immunodeficient mice. Cancer Res. 2006; 66:613-621.
22. Lin YC, Chen HW, Kuo YC, Chang YF, Lee YJ, Hwang JJ. Therapeutic efficacy evaluation of curcumin on human oral squamous cell carcinoma xenograft using multimodalities of molecular imaging. Am J Chin Med. 2010; 38:343-358.
23. Du WZ, Feng Y, Wang XF, Piao XY, Cui YQ, Chen LC, Lei XH, Sun X, Liu X, Wang HB, Li XF, Yang DB, Sun Y, et al. Curcumin suppresses malignant glioma cells growth and induces apoptosis by inhibition of SHH/GLI1 signaling pathway *in vitro* and *in vivo*. CNS Neurosci Ther. 2013; 19:926–936.
24. Dujic J, Kippenberger S, Ramirez-Bosca A, Diaz-Alperi J, Bereiter-Hahn J, Kaufmann R, Bernd A, Hofmann M. Curcumin in combination with visible light inhibits tumor growth in a xenograft tumor model. Int J Cancer. 2009; 124:1422–1428.
25. Zhou Q, Ye M, Lu Y, Zhang H, Chen Q, Huang S, Su S. Curcumin Improves the Tumoricidal Effect of Mitomycin C by Suppressing ABCG2 Expression in Stem Cell-Like Breast Cancer Cells. PLoS One. 2015; 10:e0136694.
26. Yu J, Peng Y, Wu LC, Xie Z, Deng Y, Hughes T, He S, Mo X, Chiu M, Wang QE, He X, Liu S, Grever MR, Chan KK, Liu Z. Curcumin down-regulates DNA methyltransferase 1 and plays an anti-leukemic role in acute myeloid leukemia. PLoS One. 2013; 8:e55934.
27. Wang L, Wang L, Song R, Shen Y, Sun Y, Gu Y, Shu Y, Xu Q. Targeting sarcoplasmic/endoplasmic reticulum Ca(2)+-ATPase 2 by curcumin induces ER stress-associated apoptosis for treating human liposarcoma. Mol Cancer Ther. 2011; 10:461–471.
28. Shabtay A, Sharabani H, Barvish Z, Kafka M, Amichay D, Levy J, Sharoni Y, Uskokovic MR, Studzinski GP, Danilenko M. Synergistic antileukemic activity of carnosic acid-rich rosemary extract and the 19-nor gemini vitamin D analogue in a mouse model of systemic acute myeloid leukemia. Oncology. 2008; 75:203-214.
29. Sharabani H, Izumchenko E, Wang Q, Kreinin R, Steiner M, Barvish Z, Kafka M, Sharoni Y, Levy J, Uskokovic M, Studzinski GP, Danilenko M. Cooperative antitumor effects of vitamin $D_3$ derivatives and rosemary preparations in a mouse model of myeloid leukemia. Int J Cancer. 2006; 118:3012–3021.

## MATERIALS AND METHODS

### Determination of hematopoietic cell surface markers

Aliquots of 5 × $10^5$ cells were harvested, washed twice with PBS, and incubated for 45 min at room temperature with the following fluorophore-conjugated mouse monoclonal antibodies: CD34-APC, CD38-FITC (BioLegend, San Diego, CA, USA), CD14-FITC or CD11b-PE (eBioscience, San Diego, CA, USA). The cells were then washed three times with ice-cold PBS and resuspended in 1 ml of PBS. Analysis was performed using a Gallios (Beckman Coulter) or a FACSCanto II (BD Bioscience, San Jose, CA, USA) flow cytometer. Data processing was done by FlowJo software (FlowJo, Ashland, OR, USA). Isotype control mouse antibodies were used to set threshold parameters.

### Cell cycle analysis

Following staining with CD14 or CD34/CD38 antibodies (see "Determination of hematopoietic cell surface markers by flow cytometry", Material and Methods), 1 × $10^6$ PBMC or umbilical cord blood cells were fixed in 1% paraformaldehyde and kept at 4°C for 24 h. Cells were then washed thrice with PBS and incubated in PBS containing DNAse-free RNase (30 µg/ml) and 0.1% Triton X-100, for 60 min at room temperature. Cells were stained with propidium iodide (PI, 10 µg/ml, Sigma), for 15 min, and DNA histograms were analyzed using a FACSCanto II flow cytometer (BD Bioscience).

### Real time quantitative RT-PCR

Total RNA was purified from cell cultures using RNA extraction kit (PerfectPure RNA Tissue Kit; 5 Prime, Gaithersburg, MD, USA) according to manufacturer's protocol. RNA quantification was carried out using a micro-volume spectrophotometer (NanoDrop, Wilmington, DE, USA). First-strand cDNA was generated by reverse transcriptase kit (qScript cDNA Synthesis Kit; QUANTA Biosciences, Gaithersburg, MD, USA) using oligo(dT) and random primers. Quantitative cDNA amplification was performed in a StepOne Real-Time PCR System (Thermo Fisher Scientific, Wilmington, DE, USA) using SYBR Green master mix (PerfeCTa SYBR Green FastMix; QUANTA Biosciences). The primer sequences used in this study were as follows: SERCA2b [ATP2A2; NCBI Gene ID 488 (forward: 5′-CGCTACCTCATCTCGTCCA-3′; reverse: 5′-TCGGGTATGGGGATTCAA-3′)]; SERCA3 [ATP2A3, NCBI Gene ID 489 (forward: 5′-CTCTGACTTGCCTG GTGGAG-3′; reverse: 5′-GGTGAACTCCTTCCGCA TCA-3′). Beta-glucuronidase (GUSB; NCBI Gene ID 2990) was used as a reference gene (forward: 5′-GTGGTGCG TAGGGACAAGAAC-′; reverse: 5′-TGAAGATGGTGAT CGCTCACAC-3′).

### Measurements of mitochondrial $Ca^{2+}$ uptake by flow cytometry

Cells (0.6 × $10^5$/ml) were suspended in 0.5 ml $Ca^{2+}$-supplemented (2 mM) Ringer's solution containing 10 µM Rhod-2/AM and 0.1% BSA, incubated in the dark for 30 min at 37ºC, washed and re-suspended in $Ca^{2+}$-supplemented Ringer's solution. Rhod2 fluorescence was monitored as a function of time on a Gallios instrument equipped with the 561 nm excitation laser (Beckman Coulter), before (resting values) and after the addition of 10 µM thapsigargin to cell suspension. Data were processed with FlowJo software (FlowJo, Ashland, OR, USA) and exported to Microsoft Excel, followed by normalization of the instantaneous changes in Fluo-3 fluorescence to the resting values. Rates of mitochondrial $Ca^{2+}$ uptake were expressed as slopes of initial 30-sec curve fragments after beginning of mitochondrial $Ca^{2+}$ ($Ca^{2+}_{mit}$) rise.

## REFERENCES


1. Hao QL, Shah AJ, Thiemann FT, Smogorzewska EM, Crooks GM. A functional comparison of CD34+ CD38- cells in cord blood and bone marrow. Blood. 1995; 86:3745–3753.
